# The kisspeptin analog C6 reverses reproductive dysfunction in a mouse model of hyperprolactinemia

**DOI:** 10.1530/REP-25-0036

**Published:** 2025-03-17

**Authors:** Chloe Beaudou, Louise Sionneau, Didier Lomet, Vincent Robert, Peggy Jarrier Gaillard, Vincent Aucagne, Hugues Dardente, Massimiliano Beltramo, Vincent Hellier

**Affiliations:** ^1^ INRAE, CNRS, Université de Tours, PRC, Nouzilly, France; ^2^ Centre de Biophysique Moléculaire, CNRS UPR 4301, Orléans, France

**Keywords:** reproduction, prolactin, kisspeptin, mouse

## Abstract

**In brief:**

Kisspeptin has been shown to be tightly associated with hyperprolactinemia. This study shows that similar to kisspeptin, its analog C6 produces a reversal of HPRL estrus cycle and ovulation disruption.

**Abstract:**

HPRL, characterized by elevated prolactin levels, disrupts the hypothalamic–pituitary–gonadal axis, leading to reproductive dysfunctions such as menstrual irregularities, anovulation and infertility. Current treatments rely on dopamine agonists but are limited by side effects and resistance. Kisspeptin (Kp), a key neuropeptide regulating the reproductive function, offers potential as an alternative therapy. However, Kp’s short half-life requires impractical administration regimens. To address this, we developed a synthetic Kp analog, C6, with enhanced pharmacokinetics. This study evaluated the effects of C6 compared to Kp in a mouse model of HPRL. Mice received subcutaneous PRL injections for 21 days to induce HPRL, followed by daily or alternate-day intraperitoneal administration of Kp10, C6 or vehicle. Estrous cyclicity, luteinizing hormone (LH) secretion, ovarian histology and hypothalamic gene expression were analyzed. As expected, the HPRL treatment blocked estrous activity, which was restored by both Kp10, the shortest bioactive isoform of Kp, and C6. Histological analysis revealed increased corpora lutea in Kp10- and C6-treated groups, indicating restored ovulation. C6 demonstrated equivalent efficacy to Kp10 in mitigating HPRL-induced reproductive dysfunctions, offering a promising alternative therapy. Future investigations should further explore the mechanistic advantages of C6, particularly its role in LH regulation, to optimize treatment strategies for HPRL-related reproductive disorders.

## Introduction

Hyperprolactinemia (HPRL), characterized by elevated levels of prolactin in the blood, presents a significant challenge to reproductive health, particularly in women. Prolactin (PRL), traditionally known for its role in lactation in a large variety of species (McNeilly 2001), exerts inhibitory effects on the hypothalamic–pituitary–gonadal (HPG) axis by suppressing the secretion of gonadotropin-releasing hormone (GnRH) (Koike *et al.* 1991, Selmanoff *et al.* 1991). In physiological conditions, lactation-induced elevated PRL secretion transiently inhibits the reproductive axis and prevents the occurrence of pregnancy. In pathophysiological conditions, HPRL in women leads to a long lasting inhibition of the reproductive system, characterized by reduced GnRH and LH (luteinizing hormone) secretion, menstrual irregularities, anovulation, reduced sexual desire and infertility (Bohnet *et al.* 1976). Given dopamine’s inhibitory effect on PRL release, medical treatments based on dopamine agonists (e.g., bromocriptine or cabergoline) (Krysiak & Okopień 2019) are currently the first-line of treatment for patients suffering from HPRL. However, these drugs can induce pathopsychological side effects related to compulsive behaviors, and resistance (Delgrange *et al.* 2009) can arise, thus limiting treatment efficacy.

Acting upstream of GnRH neurons, neurons that express kisspeptin (Kp), a neuropeptide encoded by the KISS1 gene, have emerged as key regulators of the HPG axis (Pinilla *et al.* 2012). Kp stimulates GnRH release, ultimately driving gonadal function. Unlike GnRH, Kp neurons do express PRL receptors (Kokay *et al.* 2011, Li *et al.* 2011, Grattan & Szawka 2019, Hackwell *et al.* 2025), and elevated levels of PRL in rodents are associated with a significant reduction of KISS1 mRNA expression (Araujo-Lopes *et al.* 2014, Hackwell *et al.* 2025). The working hypothesis is that HPRL effect on reproduction is caused by inhibition of Kp synthesis and release. This hypothesis is supported by both murine and human studies (Millar *et al.* 2017, Hackwell *et al.* 2025). Indeed, in murine models of HPRL, administration of the shortest active form of Kp composed by the ten last amino acids, called Kp10, leads to normalization of estrous cyclicity, restoration of ovulation and improved fertility (Sonigo *et al.* 2012). Similarly, in clinical studies involving HPRL patients, intravenous perfusion or subcutaneous administration of Kp resulted in enhanced GnRH secretion, normalization of menstrual cycles and improved ovulatory function (Millar *et al.* 2017, Hoskova *et al.* 2022). However, due to the short half-life of the endogenous peptide (Jayasena *et al.* 2011), a single dose of Kp is insufficient and either perfusion or multiple injections are required to achieve an effect. Such a dosing regimen is unsuitable for clinical applications. To palliate this issue, we developed a synthetic analog of Kp10, called C6, retaining high affinity for the Kp receptor (KISS1R) and with enhanced pharmacokinetic properties compared to native Kp10 (Decourt *et al.* 2016). This analog has been shown to be active in several species (Decourt *et al.* 2016, 2019, Parker *et al.* 2019, Fanelli *et al.* 2022, Salzano *et al.* 2022) and could represent a potential alternative, or adjunctive therapy, for HPRL-associated reproductive dysfunctions. However, C6 effect on HPRL has not been tested yet. The aim of this study was to establish the possible advantage of C6 compared to Kp in rescuing reproductive function in a mouse model of HPRL. Treatments have been administered daily or every other day in a mouse model of HPRL and estrus cyclicity, LH plasma level and ovarian histology were used as readouts for efficacy. Overall, we show here that HPRL-induced reproductive dysfunction could potentially be reversed by C6 treatment and thus represent an interesting advance for possible therapeutic application.

## Materials and methods

### Mice and treatments

All procedures were performed according to the EU legislation, were approved by the local ethical committee (Ethical Committee for Animal Experimentation Val de Loire) and authorized by the French Ministry ‘de l’enseignement supérieur et de la Recherche’ (authorization no. 2022101316093104). Nine-week-old C57Bl6/J mice (*Mus musculus*) of approximatively 20 g were housed four per cage, with free access to food and water under a 12 h light:12 h darkness cycle (from 07:00 h to 19:00 h). After a period of habituation, they received twice daily a subcutaneous (s.c.) injection of prolactin (NIDDK-oPRL-19, AFP-9221A) at a dose of 7 μg/day (dissolved in 18% ethanol 100% in phosphate buffer 0.1 M; HPRL group) or vehicle (18%Eth/PB) (Control group) during 21 days. Control and HPRL mice groups were further subdivided in three different groups receiving daily intraperitoneal (i.p.) injections of either mouse Kp10 (1 nmol, GeneCust, France), C6 (1 nmol, synthesized in the laboratory) or DMSO (1% in saline solution, D4540 Merck), the vehicle of Kp10 and C6 solutions. Kp10- and C6-treated HPRL groups were subdivided in two additional groups receiving every 2 days an i.p. injection of either Kp10 (1 nmol) or C6 (1 nmol). Kp, C6 or DMSO treatments started on the same day as PRL injections.

### Analysis of estrous cyclicity

The estrous cycle stage of female mice was determined by vaginal smear following the method described by McLean and coworkers (McLean *et al.* 2012). Smears were taken each day during 20 days, approximately 2 h after the beginning of the light period. Analysis of the presence of either lymphocytes, nucleated epithelial cells or keratinocytes was performed in a blinded manner by the same experimenter that assigned the status of the mouse to one of the different estrous phases: diestrus, metestrus, proestrus and estrus.

### LH ELISA

After the 21 days experimental period of the protocol, at 09:00 h, blood samples (4 µL) were collected every 20 min during 2 h starting at 09:00 h and lasted until 11:00 h after an initial and unique section of 1 mm of the tail (Steyn *et al.* 2013). Blood samples were collected immediately after Kp10, C6 or vehicle administration for the daily-treated groups and after vehicle administration for 2-day treatment group. A sandwich ELISA, adapted from Steyn and coworkers (Steyn *et al.* 2013) and previously described (Decourt *et al.* 2016), was used to measure LH concentration in blood. Monoclonal bovine LHβ 518 B7 (1:1,000 dilution) was used as the capture antibody (obtained from Lillian Sibley at University of California Davis), and the standard was NIDDK mouse LH reference preparation from AF Parlow (AFP5306A mouse RIA kit). The assay sensitivity was 0.2 ng/mL and the mean intra-assay coefficient of variation averaged 11%. The individual analyses of all samples collected were reported as the area under the curve (AUC) of the LH profile of each animal.

### Prolactin ELISA

Twenty-two days after the beginning of the protocol, blood samples were collected after decapitation and PRL measured by ELISA method (E0022Sh, BT Lab), following the manufacturer’s instructions. The assay sensitivity was 2.54 ng/mL. Mice receiving 7 μg/day injections of PRL induced a PRL serum concentration of 225.6 ± 9.9 ng/mL.

### Ovaries treatment

Twenty-two days after the beginning of PRL treatment, mice were decapitated and ovaries were sampled. Samples were immersed in PBS and fixed for 4 h in 4% PFA, washed, paraffin-embedded and sectioned at 4 µm. Sections were stained with hematoxylin and eosin Y. All sections were collected and images acquired with an AxioScanZ1 slide scanner (Carl Zeiss, France). Both ovaries of each animal were examined and the number of corpora lutea counted in all ovaries.

### Statistics

Data were analyzed using the GraphPad Prism 10.2.3 and are expressed as box and whiskers. Differences between groups were analyzed using the Kruskal–Wallis statistical test, followed by Dunn’s post-hoc test. *P* values lower than 0.05 were considered statistically significant.

## Results

**Figure 1:**
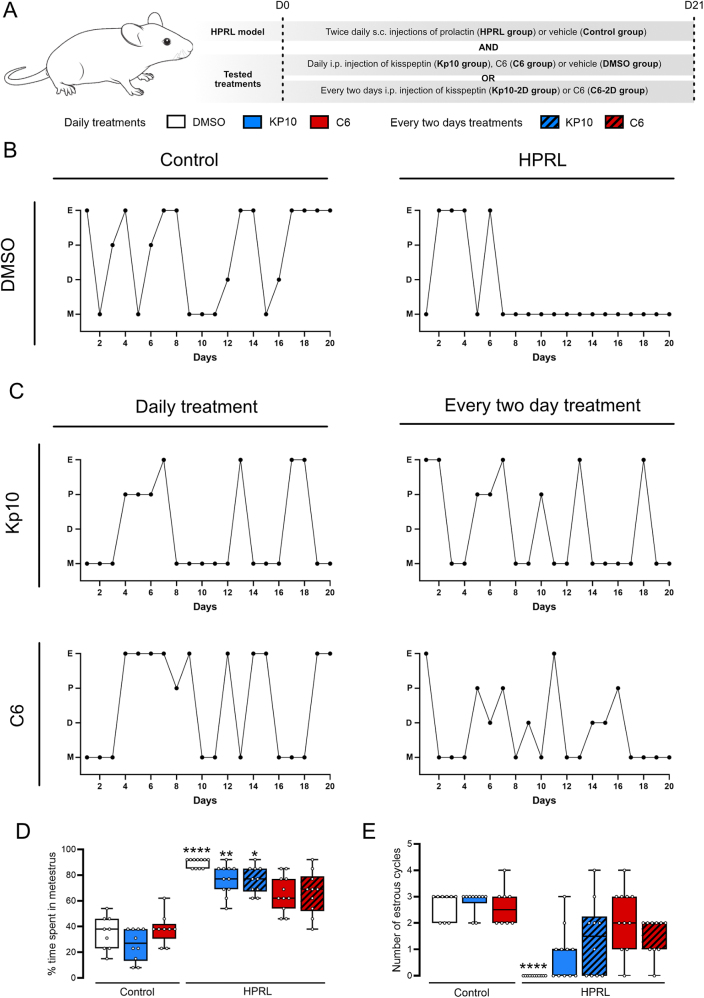
HPRL mouse model and effect of treatments on estrous cyclicity. (A) Mice are receiving twice a day an injection of prolactin at a dose of 7 μg/day (HPRL group) versus vehicle (Control group). An additional treatment is administered daily (Kp10, C6 or DMSO group) or every 2 days (Kp10-2D or C6-2D group) in order to evaluate the effect of Kp10 and C6 treatment. (B, C) Representative graphs of estrous cyclicity in the Control and HPRL groups (B) and upon treatments with Kp10 or C6 (C). (D) Percentage of time spent in the metestrus phase of the estrus cycle. (E) Number of estrous cycles per group after the 7th day of treatment. Data are represented as box and whiskers. *n* = between 9 and 11 per group; data are compared to control daily vehicle (DMSO)-treated group: *P < 0.05; **P < 0.01; ****P < 0.0001 (Kruskal–Wallis test followed by a Dunn’s post-hoc analysis). 2D, 2-days; D, diestrus; E, estrus; HPRL, hyperprolactinemia; Kp10, kisspeptin 10; M, metestrus; P, proestrus.

To address the effects of Kp or C6 treatment on HPRL syndrome, we developed a HPRL mouse model by injecting PRL subcutaneously twice daily during 21 days (Fig. 1A). In mice, an estrus cycle is characterized by the succession of four phases: metestrus (24 h), proestrus (less than 24 h), estrus (less than 24 h) and diestrus (approximately 2 days) (Cora *et al.* 2015). As expected, control mice showed 4–5 days regular estrus cycles, while HPRL mice, starting from the 7th day of treatment and until the end of the injection period (21 days), exhibited abnormal or absent estrus cyclicity (Fig. 1B and Supplementary Figure 1 (see section on Supplementary materials given at the end of the article)). However, daily or every 2 days injection of Kp10 or C6 induced an improvement of the regularity of the estrus cyclicity (Fig. 1C and Supplementary Figure 1). Overall, HPRL mice spent significantly more time in metestrus compared to control mice (*P* < 0.0001) and exhibited less estrus phase (Fig. 1). Interestingly, the percentage of time spent in metestrus by HPRL mice upon C6 treatment (either injected daily or every 2 days) is not significantly different from control mice (*P* = 0.2792 and *P* = 0.2560, respectively). Conversely, Kp-10-treated mice spent significantly more time in metestrus compared to control DMSO-treated mice (daily injection: *P* = 0.008; every 2 days injection *P* = 0.01) (Fig. 1D). However, the number of estrous cycles (defined by a transition between metestrus/diestrus and estrus) between HPRL group and HPRL-Kp10- or C6-treated groups (either injected daily or every 2 days) was not statistically different. Both Kp10- and C6-treated mice showed an improvement of estrus cyclicity with an increase in the number of estrous cycles, compared to HPRL mice treated with vehicle (Fig. 1E).

**Figure 2:**
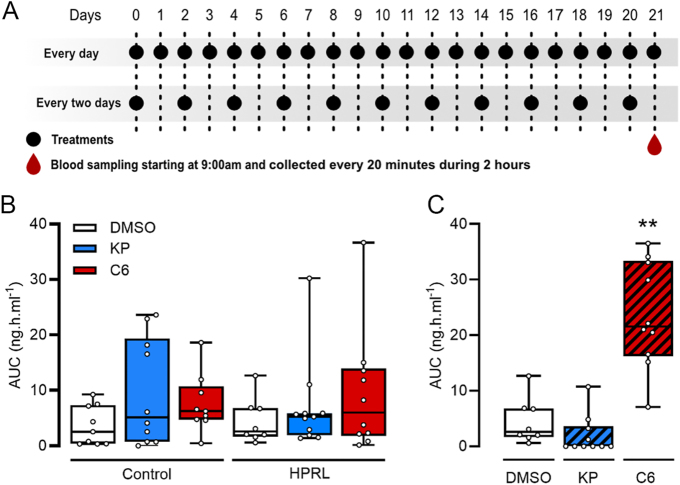
Effect of Kp10 and C6 treatments on LH secretion. (A) Schematic representation of the 21 days (one dotted line representing 1 day) of treatment, with an administration every days or every 2 days (black dots) and serial blood sampling over 120 min in HPRL and control mice at the end of the 21 days upon administration of the different treatments. (B, C) Evaluation of the total amount of LH secreted (AUC) upon DMSO, Kp or C6 administration everyday (B) or every 2 days (C). Results are represented as AUC as box and whiskers. *n* = between 9 and 11 per group. Data are compared to control vehicle (DMSO)-treated group: **P < 0.01 (Kruskal–Wallis test followed by a Dunn’s post-hoc test).

At the end of the 21-day treatment period, Kp10 or C6 have been injected to evaluate the effect of chronic treatments on LH secretion after bolus injection (Fig. 2A). Of note, daily treated mice received an injection of respective treatments on the day of blood sampling. Every 2 days treated mice were injected the day before the blood sampling. Blood sampling was performed every 20 min during 2 h. Daily injections of both treatments did not affect LH secretion in control mice (Fig. 2B). However, C6 (but not Kp10) injection every 2 days had a significant effect on LH secretion of HPRL mice in comparison to control HPRL mice (*P* = 0.0087) (Fig. 2C).

**Figure 3:**
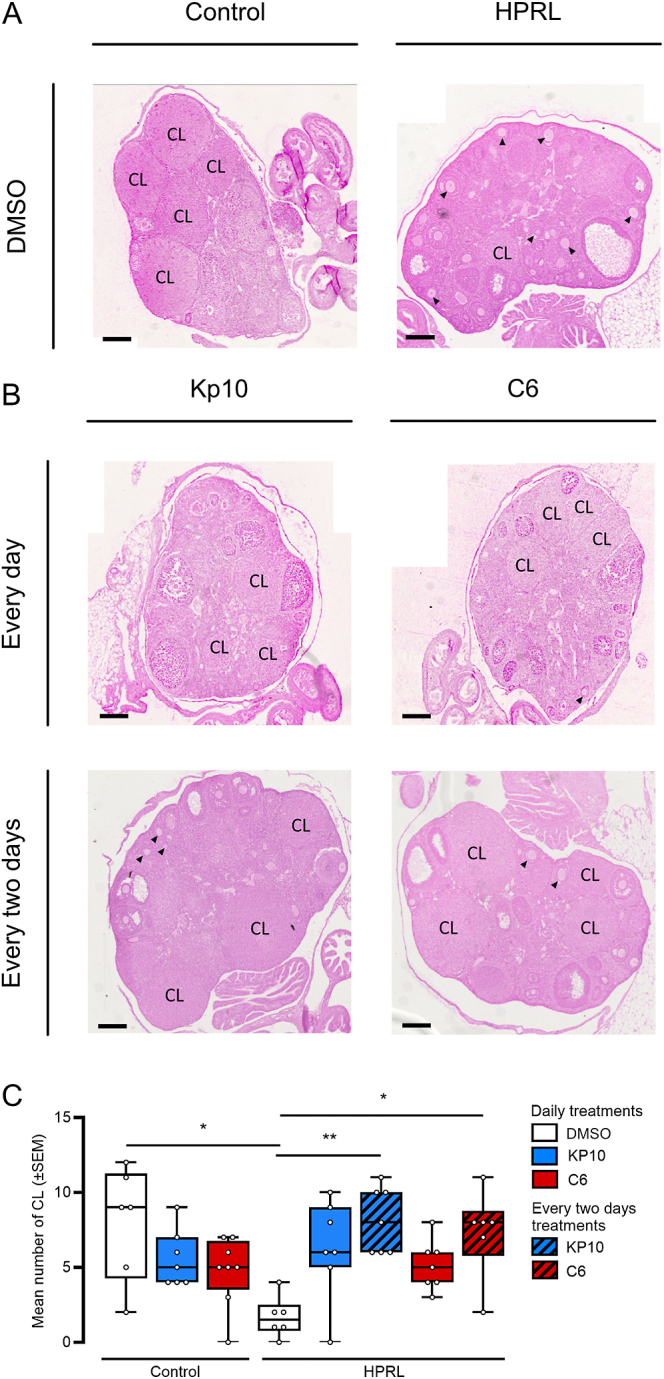
Ovarian histology (A, B) and number of corpora lutea (C) upon Kp10 and C6 treatment on HPRL mice. Arrows in A and B indicate primary follicles. Scale bar 150 μm. Abbreviations: CL, corpus luteum; HPRL, hyperprolactinemia; KP10, kisspeptin 10. *n* = between 6 and 8 per group *P < 0.05; **P < 0.01 Kruskal–Wallis followed by a Dunn’s post-hoc test.

To gain further insight into the effect of Kp10 and C6 treatment on the reproductive function of HPRL mice, ovaries from HPRL and control mice were collected and analyzed. As shown in Fig. 3, HPRL mice showed a significant reduction of the number of corpora lutea as compared to control mice (*P* = 0.0180; Kruskal–Wallis). Of note, histological observation of the ovaries of HPRL mice revealed the presence of numerous primary follicles (Fig. 3A and B). In HPRL mice, Kp10 or C6 treatments provided every other day, but not daily, increased the number of corpora lutea compared to control HPRL mice (*P* = 0.0044 and *P* = 0.0326, respectively) (Fig. 3B and C).

## Discussion

Here, we show that C6, a Kp analog developed in our laboratory, reduced the negative reproductive impact of PRL in a mouse model of HPRL. As previously reported (Sonigo *et al.* 2012), Kp10 treatment restored estrus cyclicity over a 21 days period. C6 treatment elicited a similar effect. Under a protocol of PRL daily administration, both compounds, regardless of administration frequency, improved estrous cyclicity (decreasing the time spent in metestrus and increasing the number of estrus cycle, designated by a mestestrus/diestrus to estrus phase transition). At the beginning of the treatment protocol, the stage of the estrus cycle has not been considered. In this context, given the potential luteotrophic (Gibori & Richards 1978) effect of prolactin, our treatment regimen could have induced pseudopregnancies, resulting in the variations observed in the estrus cyclicity of some animals (Supplement Fig. 1). As a biomarker of reproductive physiology status, we analyzed that the ability of the reproductive system respond to Kp or through assessment of LH secretion. To this end, blood sampling was performed in the morning. Acute injection of C6 in the afternoon triggers an LH preovulatory surge (Decourt *et al.* 2016), which might complicate the interpretation of our data. It would have been interesting to perform this test during the afternoon to check if Kp10 or C6 would induce an ovulatory surge, but this would not have been possible using the same experimental groups. Hence, we decided at first to check if C6 had an effect on LH secretion. It would be interesting to investigate this aspect in future research. Control and HPRL mice exhibited low LH levels, which are consistent with a lack of stimulation on one hand and inhibition induced by PRL administrations on the other hand. At the end of the daily treatment period, neither Kp nor C6 injection elicited a significant increase of LH blood level. Clinical trials in women suffering from reproductive dysfunction and treated twice daily with Kp showed that such a pattern of administration led to receptor desensitization and an interruption of the stimulatory effect of Kp on gonadotropin secretion (Jayasena *et al.* 2009). Additional experiments led in sheep revealed that continuous administration of Kp prevented the effect of the peptide on LH secretion over time, which is in accordance with a desensitization of the receptor (Caraty *et al.* 2007). Finally, studies in male rats revealed similar LH releasing profiles upon continuous subcutaneous administration, with a reduction of LH secretion within 24 h (Roa *et al.* 2008, Matsui *et al.* 2012). Taken together, these observations suggest that daily administration of Kp or C6 in our protocol could potentially lead to receptor desensitization and an absence of stimulation of LH release.

More interestingly, every 2 days administration of Kp or C6 led to divergent effects on LH release. C6-treated mice showed an elevated LH level, whereas in Kp-treated mice LH, levels were similar to that of the control group. The different half-life of Kp10 and C6 could help explain this result. Indeed, with a very short half-life (Jayasena *et al.* 2011), every 2 days injection of Kp may not be sufficient to have a long lasting effect on LH release. However, C6, with a half-life of several hours, seems to induce a marked increase of LH levels in the plasma. This may be due to the capacity to reach a steady state concentration of C6 that is not inducing receptor desensitization. However, continuous LH release is not ideal to induce physiological stimulation of ovulation. Hence, a different dosing regimen such as a 3 or 4 days injection may be more appropriate to prevent this supraphysiological stimulation of LH release observed in our study.

To characterize further the impact of the treatments, we performed histological analysis of ovaries. Overall, treatments with Kp and C6 induced a significant increase in the number of corpora lutea compared to HPRL mice control group. In contrast to the effect on LH release, daily and every 2 days treatments with either molecule elicited the same effect on ovulation and mirrored the effects observed on estrus cyclicity. These results suggest a potential dichotomy between central versus peripheral effect of the treatments. In line with this hypothesis, recent findings revealed a potential direct effect of Kp on the ovaries. Indeed, expression of the Kiss1 receptor has been detected in the ovary of several species (Saadeldin *et al.* 2012, Dorfman *et al.* 2014, Cielesh *et al.* 2017) and oocyte-selective ablation of Kiss1R leads to ovarian failure without significant effect on puberty onset (Dorfman *et al.* 2014, Ruohonen *et al.* 2022). Although these studies have not been performed with C6 yet, an ovarian effect of C6 cannot be excluded. Hence, our results may be explained by a distinct direct action of Kp and C6 in the ovaries, which could lead to a stimulation of ovulation.

Due to the central role of Kp in reproduction, pharmacological regulation of the female HPG using Kp or its analog C6 is of high interest. Interestingly, the endogenous Kp10 peptide acts upon different parameters of the reproductive physiology, from ovulation to sexual desire and behavior (Hellier *et al.* 2018, Thurston *et al.* 2022), which presents substantial therapeutic potential in a wide range of reproductive disorders. To overcome biological limitation of short-lived Kp10 activity, we developed C6, an analog with similar effect on puberty, ovulation and LH release in various species (Decourt *et al.* 2016, 2019, Parker *et al.* 2019, Fanelli *et al.* 2022, Salzano *et al.* 2022), but with improved half-life. We show that, similar to Kp10, C6 produces a reversal of HPRL ovarian cycle disruption. In addition, depending on the dosing regimen, it has an additional effect on LH secretion. The potential advantage of this effect on LH should be further investigated to fully understand its usefulness in the treatment of HPRL.

## Supplementary materials



## Declaration of interest

M Beltramo and V Aucagne are inventors in a patent on C6 held by INRAE.All other authors declare that there is no conflict of interest that could be perceived as prejudicing the impartiality of the research reported

## Funding

This work did not receive any specific grant from any funding agency in the public, commercial or not-for-profit sector.

## Author contribution statement

V Hellier helped in writing of the original draft, review and editing, project administration, investigation, funding acquisition, formal analysis and conceptualization. C Beaudou and L Sionneau helped in writing, investigation and data curation. D Lomet, V Robert and P J Gaillard helped in investigation. V Aucagne and H Dardente helped in writing of the review and editing. M Beltramo helped in funding acquisition, conceptualization, writing of the review and editing.

## Data availability

Data will be made available on reasonable request to the corresponding author.
