# Supplementary material for: The kisspeptin analog C6 reverses reproductive dysfunction in a mouse model of hyperprolactinemia
Source: Reproduction. 2025 Mar 17;169(4):e250036. doi: 10.1530/REP-25-0036 (PMC11949517; doi:10.1530/REP-25-0036)

Supplementary Figure 1. Representative graphs of estrous cyclicity in the different experimental groups. Abbreviations: D, Diestrus; E, Estrus; HPRL, Hyperprolactinemia; Kp10, Kisspeptin 10; M, Metestrus; P, Proestrus.

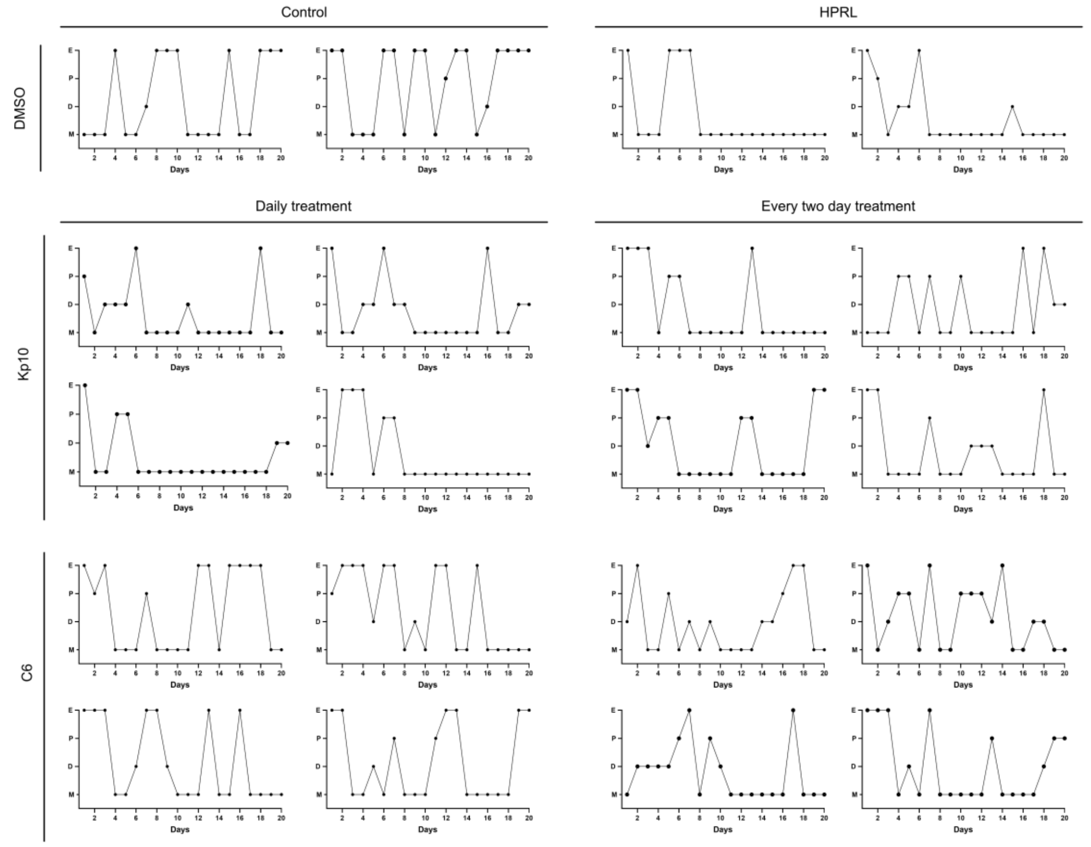

Supplement: Supplementary file 1 [file supplementary_materials.pdf]
